# Study on Structure and Properties of La_2_O_3_-Doped Basaltic Glasses for Immobilizing Simulated Lanthanides

**DOI:** 10.3390/ma14164709

**Published:** 2021-08-20

**Authors:** Qin Tong, Jichuan Huo, Xingquan Zhang, Zhu Cui, Yongchang Zhu

**Affiliations:** 1 State Key Laboratory of Environment-Friendly Energy Materials, Southwest University of Science and Technology, Mianyang 621010, China; tongqin518@163.com; 2 Electromechanic Engineering College, Mianyang Teachers’ College, Mianyang 621000, China; 3 Fundamental Science on Nuclear Wastes and Environmental Safety Laboratory, Southwest University of Science and Technology, Mianyang 621010, China; zyc_qy@163.com; 4 Analysis and Testing Center, Southwest University of Science and Technology, Mianyang 621010, China; 5 Ceramics Science Institute, China Building Materials Academy, Beijing 100024, China; 13520204133@139.com

**Keywords:** La_2_O_3_-doped, basaltic glass, HLW, PCT

## Abstract

The La_2_O_3_-doped basaltic glass simulated high-level waste form (HLW) was prepared by the solid-state melt method. The simulated waste La_2_O_3_ maximum loading and the doping effect on structure, thermal stability, leaching behavior, density, and hardness of basaltic glasses were studied. XRD and SEM results show that the simulated waste loading of La_2_O_3_ in basaltic glass can be up to ~46 wt.%, and apatite (CaLa_4_(SiO_4_)_3_O) precipitates when the content of La_2_O_3_ reaches 56 wt.%. Raman results indicate that the addition of La_2_O_3_ breaks the Si–O–Si bond of large-membered and four-membered, but the number of A1^3+^ involved in the formation of the network increase. Low content of La_2_O_3_ can help to repair the glass network, but it destroys the network as above 26 wt.%. DSC results show the thermal stability of simulated waste forms first increases and then decreases with the increase of La_2_O_3_ content. With the increase of La_2_O_3_ content, the density of the simulated waste form increases, and the hardness decreases. The leaching chemical stability of samples was evaluated by the ASTM Product Consistency Test (PCT) Method, which show that all the samples have good chemical stability. The leaching rates of La and Fe are three orders of magnitude lower than those of the other elements. Among them, L36 has the best comprehensive leaching performance.

## 1. Introduction

The disposal of high-level nuclear wastes (HLW) coming from the reprocessing of spent nuclear fuel is still an international problem, which is characterized by strong radioactivity, long half-life and tremendous harm. Vitrification has been considered to be one of the most effective methods for immobilization radioactive wastes, especially HLW, and it is the only industrialized curing technology in the world [1,2,3]. High Level Liquid Waste (HLLW) from the reprocessing of spent fuel is rich in dozens of elements [4,5,6], among which lanthanides make up a significant proportion. Lanthanide elements with special electron shell structure have great influence on the structure and properties of glass [7,8]. Therefore, as a typical representative of lanthanide elements, it is of great significance to study the effect of lanthanum content on the structure and properties of glass.

Basalt has existed for more than 100 million years in nature with holocrystalline texture, hypocrystalline texture and glassy texture, which has stable chemical and physical properties and strong weathering resistance [9]. Through the high temperature melting in nature, basalt has a good ability to form glass network [10]. Natural basaltic rocks contain a small amount of radioactive elements (such as uranium and thorium), which have been stable for billions of years. Therefore, the glass and glass-ceramics waste forms prepared with natural basaltic rocks can be considered as a suitable waste form for HLW immobilization. Immobilizing HLW with basaltic glass-ceramics was studied in the United States last century, which is used to deal with HLW and transuranic waste from commercial and military production, including nuclear waste from the Idaho Chemical Plant and the Savannah Plant and zeolite used to purify reactor core waste from the Three Mile Island accident [4]. Therefore, basalt is expected to be a new generation of host materials for HLW.

At present, there have been some researches on La_2_O_3_-doped basaltic glasses in the world. Feng et al. proved La, Al, and Na are exclusively bonded to PO_4_ groups and no bond was formed between these positive ions by NMR [11]. Bin et al. studied the structure of lanthanum-iron phosphate glasses by Raman and found a small amount of La_2_O_3_ couldn’t change the structure of the glass network and La^3+^ can enhance the stability of glass structures [12]. Sheng et al. found the degree of polymerization of glass first increases and then decreases with the increase of doping content of La_2_O_3_ [13]. Based on the existing literature, it can be considered that the content of La_2_O_3_ has a great influence on the structure and properties of glass [14,15,16]. But the study of La_2_O_3_-doped basaltic glasses is rare. So, through some research in this paper, it hopes to make some contributions to the long and in-depth study on basaltic glasses as host materials for HLW.

## 2. Experimental Method

### 2.1. Fabrication of Simulated Waste Glass

The components of the basaltic glass (Sichuan Aerospace Tuoxin Basalt Industrial CO., Ltd., Chengdu, China) were listed in Table 1. The designed samples were labeled as LX (X = 0, 8, 16, 26, 36, 46, 56) based on the mass ratio of La_2_O_3_ (MACKLIN, 99.99%) (0%, 8%, 16%, 26%, 36%, 46%, and 56%, respectively). The raw materials were mixed by ball-milling (QM-3SP2, Planetary ball mill, Nanjing NanDa Instrument Plant, Nanjing, China) for 30 min. About 30 g of the mixtures were heated in a corundum crucible in air at 1450 °C for 3 h to form homogeneous melts and then annealed at 600 °C for 1 h, followed by cooling to room temperature.

### 2.2. Characterization

X-ray diffraction (XRD, PANalytical Company, Eindhoven, The Netherlands) data was collected on an X’ Pert PRO diffractometer using Cu-Kα radiation (λ = 1.54187 Å). The microstructure and microtopography of the samples were studied using a Carl Zeiss Ultra 55 field emission scanning electron microscope (FE-SEM) with energy dispersive X-ray spectroscopy (EDS), and the flat block samples were corroded with 10% HF and sprayed with gold. Raman spectra was collected to characterize the vibration of the characteristic functional group of the samples using laser Raman spectrometer with CCD detector (InVia, Renishaw, UK). Spectra were acquired in the 200–2000 cm^−1^ range. The glass powders of particle size less than 200 mesh were measured using differential scanning calorimetry (DSC SDT Q600, TA Instruments Inc., New Castle, DE, USA) to determine glass transition temperature (*T*_g_), the crystallization peak temperature *T*_p_ and incipient crystallization temperatures (*T*_c_) from room temperature to room temperature-1300 °C at a rate of 10 °C/min in air. Bulk densities of the samples were determined using Archimedes method. Vickers hardness of the samples were measured using microhardness tester (TMVS-1S, Auto-instrument CO., Ltd., Kejing, Shenyang, China) with loading 100 g and charging for 10 s.

### 2.3. Chemical Stability

The leaching chemical stability of the obtained samples was evaluated by the ASTM Product Consistency Test (PCT) Method [17]. The powder samples sieved between 100 and 200 meshes cleaned with deionized water and dried. Then 1.5 g of sample was soaked in 15 mL deionized water in Teflon reactors and kept in an oven at 90 ± 2 °C. Leachates were taken from the reactors at the end of 1, 3, 7, 14, 28 days respectively, and then 15 mL new deionized water was added. The ion concentrations (Si, Ca, Al, Fe, La) in leachate were obtained by inductively coupled plasma mass spectrometry (ICP-MS, 7700X, Agilent Technologies, Santa Clara, CA, USA). The normalized elemental leaching rate *LR*_i_ (g·m^−2^·d^−1^) can be expressed by the following Equation (1):

(1) LRi=CifiSVt

where *C*_i_ is the concentration of the i-th element in solution (g·L^−1^), *f*_i_ is the mass fraction of the i-th element in the sample, *V* is the volume of the solution (L), *S* is the powered samples surface area (m^2^), *t* refers to the soaking time (d).

## 3. Results and Discussion

### 3.1. Glass and Crystallization Behavior

**Figure 1:**
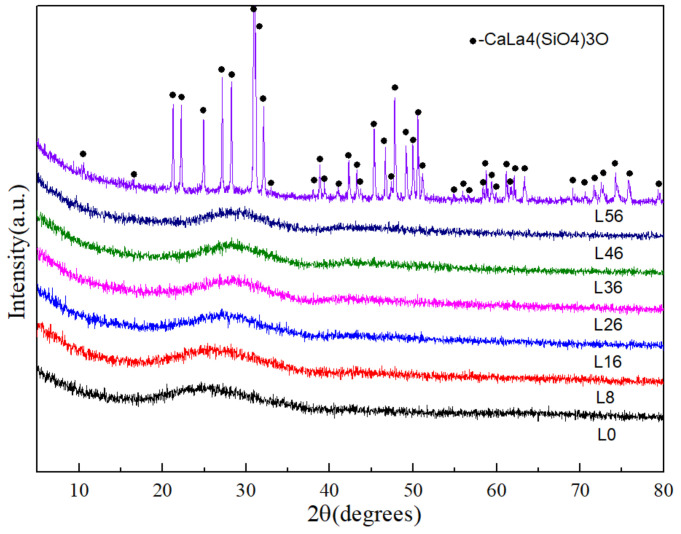
XRD patterns from L0 to L56.

Figure 1 illustrates XRD patterns of the L0-L56. When L = 0–46 in LX, it is glassy, however, all of the diffraction peaks are consistent with apatite (CaLa_4_(SiO_4_)_3_O; PDF#71-1368) with L56. It indicates that the loading of La_2_O_3_ in basaltic glass is up to 46 wt.%.

### 3.2. SEM and EDX Analysis

**Figure 2:**
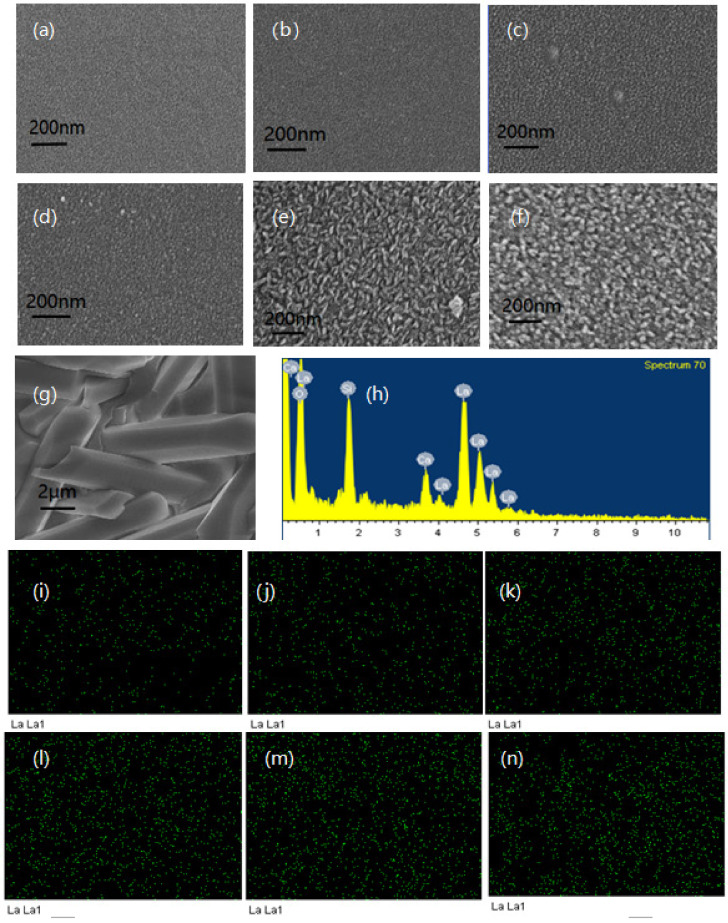
(**a**–**g**) SEM image of L0-L56. (**h**) EDX spectra under the (**g**) image region. (**i**–**n**) Elemental mapping of La from L8–L56.

Figure 2 shows SEM images of the basaltic glasses L0–L56 (a–g), elemental mapping of La from L8–L56 (h–m) and EDX spectra under the (g) image region (n). Figure 2a–f shows the crack shape of the glasses become more and more obvious with the increase of La_2_O_3_ content, which may be attributed to the effect of La_2_O_3_ on the glass structure. And, it is also obvious that there are no crystals in the basaltic glasses L0–L46, which is consistent with the XRD results. Figure 2g shows the rod-shaped crystals with a hexagonal cross section were found in the sample of L56. According to the EDX spectra as shown in Figure 2h, the precipitated crystalline phases should be consistent with the results of phase analysis. It shows that the distribution of La is basically uniform from Figure 2i–n, indicating La solidifies well in basaltic glass.

### 3.3. Raman Analysis

**Figure 3:**
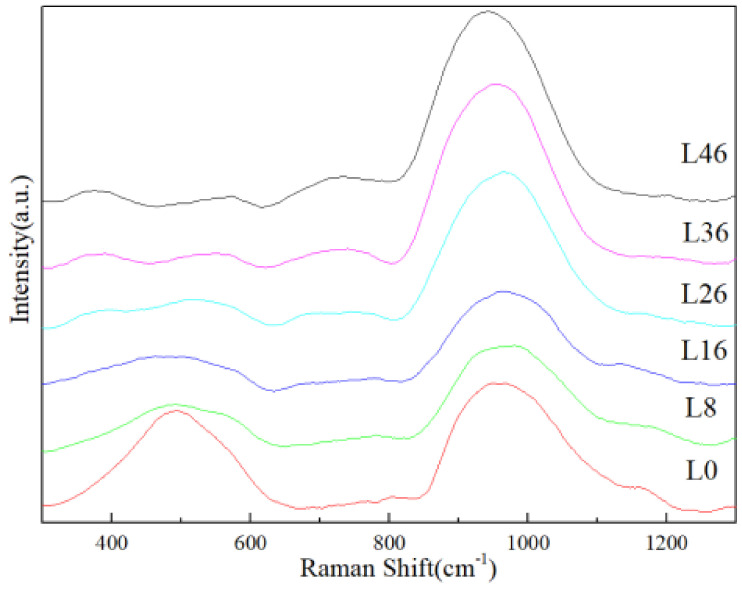
Raman spectra of basaltic glasses from L0 to L46.

In order to explore how the shape of Raman spectra varies with glass composition, we compared selected representative spectra from samples covering the entire compositional range (Figure 3). The Raman spectra of basaltic glasses can be divided into three bands: the low wavenumber (LW) band (400–650 cm^−1^), the middle wavenumber (MW) band (650–850 cm^−1^), and the high wavenumber (HW) band (850–1200 cm^−1^).

The LW band is attributed to low energy bending vibrations of bridging oxygens in a T–O–T configuration, or to angular deformation of TO_4_ groups in a O–T–O configuration, where T = Si, Al and Fe, in tetrahedral rings [18,19,20,21,22]. The characteristic peaks located at 442, 492, and 604 cm^−1^ are attributed to mixed stretching–bending vibrational modes of Si–O–Si in large-membered (≥5), four-membered, and three membered rings, respectively [23]. Figure 3 shows that the peak of L0 in the low wavenumber is mainly located at 500 cm^−1^, indicating the main role of Si–O–Si vibration of the four-membered ring. With the increase of La_2_O_3_ content, the peak gradually recedes and changes to two peaks at 400 cm^−1^ and 600 cm^−1^, indicating the addition of La_2_O_3_ breaks the Si–O–Si bond of large-membered and four-membered. The peak at 400 cm^−1^ and 600 cm^−1^ should be related to Al and Fe [18,20].

The Raman peak at the MW band (650–850 cm^−1^) is ascribed to symmetrical stretching vibration of Al-NBO (non-bridging oxygen) [20,23]. The enhancement of peak intensity indicates that the number of A1^3+^ involved in the formation of the network increase.

The HW band (850–1200 cm^−1^) is attributed to the vibrations of T–O^–^ bonds-where O^−^ stands for BO (bridging oxygen) or NBO [24]. The more bridge oxygens T is connected to, the higher wave number of T–O^–^ vibration moves to, otherwise, the fewer oxygens it’s connected to [25]. Figure 3 shows the peak moves slightly towards the high wave number from L0–L26, when the content of La_2_O_3_ exceeds 26 wt.% (L26–L46), the peak position moves slightly to the low wave number, but it doesn’t change much. This is also indirectly stated although La_2_O_3_ breaks the Si–O–Si bond, Al^3+^ is more involved in the network connection. Meanwhile, the slight shift of the peak indicates that the glass network is repaired first with the addition of La_2_O_3_ content, and the glass network is destroyed when the content of La_2_O_3_ exceeds 26 wt.%. This may be attributed to the fact that although the addition of La_2_O_3_ breaks the Si–O–Si bond, when the La_2_O_3_ content is low, La with high field intensity can enter the gap of the glass network and link the anion groups as charge compensation to repair the glass network. When the content of La_2_O_3_ is too high, the NBO in the glass network increases significantly because of too much free oxygen provided by La_2_O_3_, which destroys the glass network [12,15,26,27].

### 3.4. DSC Curves

In the process of long-term deep geological disposal, the temperature of solidified waste forms will rise due to the large amounts of heat released by the decay of radioactive elements. Therefore, solidified waste forms require good thermal stability [4,28,29]. LAFI et al. proposed to use (*T*_c_ − *T*_g_) to character the thermal stability of glass [30]. They proved that the greater the (*T*_c_ − *T*_g_) value, the less likely the glass is to nucleate and crystallize, and the better the thermal stability of the glass. Saad and Poulin used parameter *S* to represent the thermal stability of glass [31]. The greater the value of thermal stability parameter *S* by the following Equation (2), the better the thermal stability of glass.

*S* = (*T*_p_ − *T*_c_) (*T*_p_ − *T*_g_)/*T*_g_ (2)

where *S* is thermal stability parameter, *T*_p_ is the crystallization peak temperature (K), *T*_c_ is incipient crystallization temperatures (K), *T*_g_ is glass transition temperature (K).

**Figure 4:**
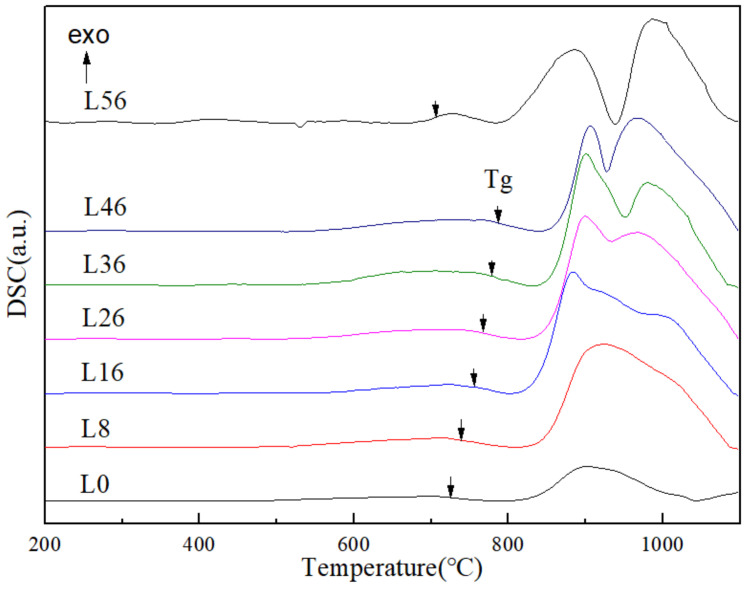
DSC curve of basaltic glasses from L0 to L56.

Figure 4 shows DSC curve of basaltic glasses from L0 to L56. Table 2 lists the glass transition temperature *T*_g_, the initial temperature of the first crystallization peak *T*_c_, the crystallization peak temperature *T*_p_ and the thermal stability parameter *S* of L0–L46. Figure 4 illustrates the glass transition temperature of the simulated waste forms increases and the crystallization peak splits from one to two with the increase of La_2_O_3_ content. It is speculated that the second crystallization peak may be related to apatite, which means there may be apatite crystals in L16–L46 that are too small to detect. The *T*_g_ increases with the increase of La_2_O_3_ content except L56, which may be related to the crystallization of L56. Table 2 indicates the thermal stability of glass-cured body firstly increases with the increase of La_2_O_3_ content, and then decreases when the content of La_2_O_3_ exceeds 8 wt.%, which may be related to changes in the glass structure. According to the previous analysis, the addition of a small amount of La_2_O_3_ is conducive to repairing the glass network, increasing the level of network connectivity and thus improving the thermal stability. However, excessive La_2_O_3_ will lead to the increase of “non-bridging oxygen” in the system which decreases the level of network connectivity and reduces the thermal stability of glass [32].

### 3.5. Density and Hardness

Table 3 shows the densities and hardness of basaltic glasses. It indicates that the densities increases and the hardness decreases with the increase of La_2_O_3_ content. As La^3+^ is located in the gap of glass network as modifier and has a higher coordination number (8 coordination), the glass network structure is more compact with the increase of La_2_O_3_ content, which shows an increase in the density of glasses macroscopically [12,33]. However, La_2_O_3_ cannot participate in the formation of the network [34]. The more La_2_O_3_ is added, the less network former exists, resulting in the loose structure of the glass network framework, which is macroscopically manifested as decreased hardness of the glass.

### 3.6. Leaching Characteristics

**Figure 5:**
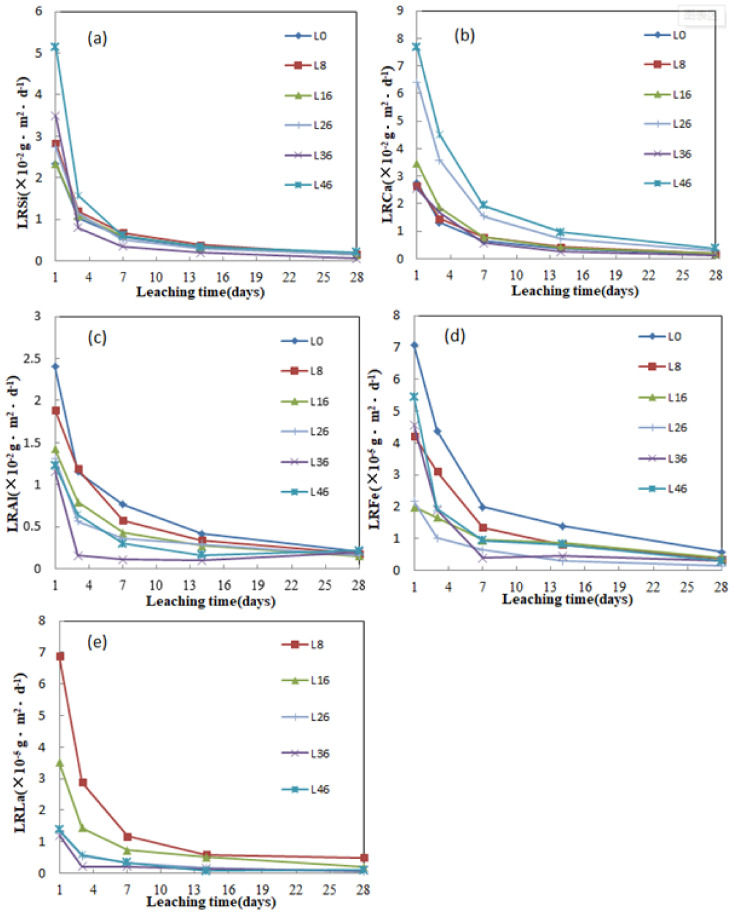
Normalized elemental leach rates of (**a**) Si, (**b**) Ca, (**c**) Al, (**d**) Fe, and (**e**) La of the L0–L46.

The chemical stability of the samples (X = 0–46 in LX) were evaluated by Product Consistency Test (PCT) Method. The computed results of the normalized leaching rates of Si, Ca, Al, Fe and La are depicted in Figure 5. It shows that the leaching rate of most samples decreased significantly with the increase of leaching time in the first 7 days, and the decreasing rate slowed down after 7 days, and basically remained unchanged after 14 days. which may be attributed to the “gel layer” hinder the leaching behavior formed by the re-polymerization of Si in solution at the interface layer of vitreous reaction with solution [35,36]. The *LR*_La_ and *LR*_Fe_ are three orders of magnitude lower than those of the other elements. After 28 d immersion, the leaching rate of each element is as low as *LR*_Si_ = 5.38 × 10^−4^ g·m^−2^·d^−1^ (L36), *LR*_Ca_ = 1.38 × 10^−3^ g·m^−2^·d^−1^ (L36) *LR*_Al_ = 1.49 × 10^−3^ g·m^−2^·d^−1^ (L16) *LR*_Fe_ = 1.3 × 10^−6^ g·m^−2^·d^−1^ (L26) *LR*_La_ = 5.82 × 10^−7^ g·m^−2^·d^−1^ (L26), respectively. It can be concluded from Figure 5a–e that L36 has the best comprehensive leaching performance, which leaching rates of Al, Fe, and La are *LR*_Al_ = 1.96 × 10^−3^ g·m^−2^·d^−1^, *LR*_Fe_ = 2.82 × 10^−6^ g·m^−2^·d^−1^, *LR*_La_ = 8.22 × 10^−7^ g·m^−2^·d^−1^, respectively. Figure 5b shows the *LR*_Ca_ of L26 and L46 is higher than that of other samples. Figure 5e shows the *LR*_La_ decreases with the increase of La content in the simulated waste forms, but it increases as above 36 wt.%. This may be attributed to the effect of La on glass structure. The leaching rates of all the samples meet the expectations of nuclear industry standards (<1 g·m^−2^·d^−1^) [37], indicating that L0–L46 has good chemical stability.

## 4. Conclusions

The La_2_O_3_-doped basaltic glass simulated waste forms were prepared by the solid-state melt method. The loading of La_2_O_3_ in the simulated waste forms is at least 46 wt.%, and apatite (CaLa_4_(SiO_4_)_3_O) is precipitated from the glass when the content of La_2_O_3_ reaches 56 wt.%. The addition of La_2_O_3_ breaks the Si–O–Si bond of large-membered and four-membered, but the number of A1^3+^ involved in the formation of the network is increasing. When La_2_O_3_ loading is low, it can help to repair the glass network with the increase of La_2_O_3_ content, but it destroys the network when above 26 wt.%. With the increase of La_2_O_3_ content, the densities of basaltic glasses increase and the hardness decreases, which may be related to the effect of adding La_2_O_3_ on glass structure and the reduction of network former. The addition of a small amount of La_2_O_3_ is beneficial to improve the thermal stability of the glass, but excessive La_2_O_3_ reduces the thermal stability of the glass. The simulated waste forms have good chemical stability, and L36 has the best comprehensive leaching performance. The *LR*_La_ and *LR*_Fe_ are three orders of magnitude lower than those of the other elements.

## Figures and Tables

**Table 1:** Components of the basaltic glass from producer.

Raw Materials	Chemical Components, wt.%								
	SiO_2_	Al_2_O_3_	Fe_2_O_3_	CaO	MgO	TiO_2_	Na_2_O	K_2_O	La_2_O_3_
L0	54.97	14.24	7.38	15.63	2.80	2.29	1.24	0.77	-
L8	50.57	13.10	6.79	14.38	2.58	2.11	1.14	0.71	8
L16	46.17	11.96	6.20	13.13	2.35	1.92	1.04	0.65	16
L26	40.68	10.54	5.46	11.57	2.07	1.69	0.92	0.57	26
L36	35.18	9.11	4.72	10.00	1.79	1.47	0.79	0.49	36
L46	29.68	7.69	3.99	8.44	1.51	1.24	0.67	0.42	46
L56	24.19	6.27	3.25	6.88	1.23	1.01	0.55	0.34	56

**Table 2:** The obtained *T*_g_, *T*_c_, *T*_p_ and *S* of L0–L46.

Sample	L0	L8	L16	L26	L36	L46
*T*_g_ (°C)	725.836	738.402	745.46	766.88	778.716	787.449
*T*_c_ (°C)	803.836	819.402	811.46	823.88	835.716	843.449
*T*_p_ (°C)	901.803	922.402	884.46	898.88	899.716	905.449
*S*	17.26	18.74	9.96	9.52	7.36	6.90

**Table 3:** Density and Vickers hardness of L0–L46.

Sample	L0	L8	L16	L26	L36	L46
Density (g/cm^3^)	2.5025	2.7504	2.9856	3.2029	3.3918	3.5034
Vickers hardness	898.52	825.80	775.84	716.42	623.04	521.20

## Data Availability

The data can be requested from the corresponding author.
